# Syphilitic Paralysis

**Published:** 1871-09

**Authors:** Lyman Ware

**Affiliations:** Chicago, Illinois


### SYPHILITIC PARALYSIS.

By LYMAN WARE, M.D., Chicago, Illinois.

It may sometimes be questionable whether it is profitable to the general practitioner to have extraordinary or rare cases written out and reported, their utility not being sufficiently apparent. Yet, in those cases which have been much neglected by our text-books, and where the pathology and consequent mode of treatment has not been fully determined upon, and where, also, prompt and definite treatment alone could substitute enjoyment for untold misery, it may not be altogether a waste of time and labor to call special attention to the study of a few cases of syphilitic disease of the nervous system which are complete in their history and typical of their kind.

Some of the earliest writers, as early even as the sixteenth century, made mention of nervous affections following far in the wake of primary syphilis. Still, there was so much of vagueness and uncertainty surrounding the cases reported, that the facts deduced from their observations were not generally accepted. So eminent a light in the medical world as John Hunter strongly denied that the brain or nervous system could be ever affected from syphilitic disease, and the celebrated Sir Astley Cooper, says, in his *Principles and Practice of Surgery*, "Some parts of the body are incapable of being acted upon by the venereal poison, as the brain, heart, and abdominal organs; indeed, the venereal poison does not appear to be capable of exercising its destructive influence on the vital organs, or those parts most essential to the welfare and continuance of life; but the bones, muscles, and skin readily partake of its malignant nature."

The positive opinion of gentlemen so highly esteemed for their profound knowledge and great skill must have blinded their cotemporaries and followers to the perception of an important truth, and retarded the recognition of a truly terrible class of maladies, otherwise, it would be unaccountable how a disease, now so fully admitted, should have been unrecognized and unacknowledged for so great length of time by men of undoubted ability and knowledge, as it is now proven by *post mortem* examinations, beyond the possibility of a doubt, that the most troublesome and dangerous tertiary symptoms are those of the nervous system.

Among the first who called special attention to syphilitic nervous diseases, giving a full account of the *post mortem* appearances, was Dr. John Watson, of New York, who published his cases in the *New York Medical Journal*, of 1843 and 1845, since which time numerous articles have appeared, both at home and abroad, corroborating the fact that syphilitic deposition may take place, either in the brain substance itself, spinal cord, or their membranes. The morbid appearances, as most commonly seen, are thickenings and adhesions of the *dura mater* to the base of the skull and brain, covered here and there with nodules, varying in size from a pea to a walnut, and of a dark yellow color. At times, these nodules are found in the brain substance itself, producing irreparable mischief, or entirely surrounding the roots of special nerves, causing local paralysis, or, seated in the cord, giving rise to complete paraplegia.

It is not yet possible to determine definitely, from *post mortem* appearance alone, syphilitic from ordinary tubercular deposit, still, by adherence carefully to the history and symptoms of the case, a line of demarcation may be drawn as distinctly as between many other diseases. Where there is syphilitic deposition, we have great and excruciating pain, particularly of a nocturnal character, with the presence of other tertiary symptoms, involving the skin, the bones, or both.

It is of the greatest importance that the true condition be early detected, and heroic specific treatment be at once adopted, else curable paralysis, *without* organic lesion, may be followed by incurable paralysis, with organic lesion. The spinal cord is less frequently affected than the brain, although precisely in the same manner. The *dura mater* of the cord being the original seat of inflammatory thickening, the exuded lymph is deposited entirely about the cord, greatly interfering with, or quite destroying its action. Interstitial inflammation and gummy tumors have been discovered in the substance of the cord, as well as in its surrounding membranes, producing complete paraplegia. In fact, paraplegia with violent sciatica, particularly on the left side, is the most common symptom of the disease in the cord. The diseases of the nervous system are gradual in their development, and those of the cord seldom make their appearance until years have elapsed after the primary lesion, which has been of either unusual violence or treatment much neglected. Loss of muscular power, or power of co-ordination, may exist, simulating progressive locomotor ataxia, from which it may be distinguished by the history and the excruciating nocturnal character of the pain in the lumbar region, extending into the loins and thigh, following the course of the sciatic nerves.

The cases given are from the private practice of the writer, and are gentlemen whose statement of facts can be fully relied upon.

The first, a case of syphilitic paraplegia, in which the exudation or deposit must have been of recent origin, judging from the rapidity with which the symptoms yielded to treatment. The second, a case in which the deposit was confined to the trunk of the *portis dura*, producing facial paralysis..

Mr. A. is a gentleman of more than usual intelligence; 34 years of age; a professional actor, and of general good health. In 1858 or 1860 he contracted syphilis. He and his mistress were subjected to treatment at the same time. Being then actively engaged, he does not remember as to the length of time he was under treatment, but thinks about a month. He suffered no inconvenience, nor did the disease in anywise show itself until the winter of 1870, ten years after inoculation, when he had a large rupial ulcer on either tibia, which was only healed upon resorting to constitutional treatment, leaving the scars and peculiar tint still well marked. As soon as the ulcers healed medication was at once discontinued. No inconvenience again occurred until the winter of 1871, when he was troubled with occasional nocturnal pains in the tibia and lumbar region, for which he received alterative treatment of iodide of potassium, keeping the pains in check, though not subdued. In April, the pains returned with much greater violence and longer duration. In fact, he was scarcely free from them at any time, although they were much worse at night, even so excruciating as to keep him awake, and oblige him to spend many sleepless nights. During the second week of June, weakness of the lower extremities began to make itself manifest, unsteadiness of gait, inability to go up or down stairs, or walk any distance. This condition gradually terminated in complete loss of motion. When I first saw him, on the 12th of June, he was not only unable to walk, but he could not even stand unsupported, and required an asssistant to turn him in bed. The lumbar pain was excruciatingly violent; the lower limbs were cold and numb, and, though movable, there was loss of co-ordination, resembling locomotor ataxia; no trouble in urinating or defecating; electro-muscular contractility, and sensibility diminished. Ordered iodide of potassium in ten-grain doses, three times daily, and a mercurial vapor bath at night; administered galvanization of the spine and lower extremities, by a current from a 32-cell battery; seance eight minutes, caused profuse perspiration.

*June 13.* Pain not so excruciating, and was able to sleep more the past night; symptoms otherwise unchanged. Treatment continued, excepting general and local faradization, which was substituted for galvanization.

*June 14.* Not so well; had more pain and less sleep than on the preceding night; medication continued, and galvanization again resorted to instead of faradization, also ordered jugs of hot water to the feet at night.

*June 15.* Patient much better; had a tolerable night's rest; galvinization employed, seance lasting ten minutes.

*June 16.* Patient decidedly better; slept well the past two nights; limbs not so cold and numb; prickly sensation much diminished. He now feels confident that he will recover. Internal treatment unchanged; vapor bath and galvanization only to be employed on alternate days. Patient went on rapidly toward recovery, so that by the latter part of the month he could walk by the aid of a cane, and on the 10th of July he could walk as erect and as firm as ever. Although he is still under treatment, it is more to prevent a recurrence of the disease, than a necessity for its continuance.

Nill says, "The course of the affection is slow, and, though it may be checked by treatment for a time, is seldom cured." Barton says of these cases, "They advance to a certain point toward recovery, but complete restoration does not take place."

Was it galvanization or early treatment that prevented so untoward a termination?

Mr. L. a sailor; age 38; had syphilis sixteen years since. Primary disease healed quickly under his own treatment, subsequently had sore throat, which rapidly healed by internal medication during a short stay at the hospital. Has had, at times, after unusual or severe exposure, "Ricord's tertiary accidents."

In 1869, fourteen years after the primary lesion, facial paralysis gradually made its appearance. When he first came under treatment, in January, he had been for the eighteen preceding months under homœopathic treatment for paralysis, but not for syphilis. The right angle of the mouth droops; the cheek hangs loose; features much distorted; diminution of sensibility not perceptible; farado-muscular contractility lost; galvano-muscular contractility normal; iritis with adhesions in the left eye from former diseases. Placed at once upon pil. hyd., with inunction of ungt. hyd. at night, and administered galvanism every second day, applying one electrode either over the mastoid process or articulation of maxilla, and the other upon the paralyzed muscles. After the second seance there was decided improvement, and after the tenth, restoration was so nearly complete that further application was omitted.

This case is particularly interesting, showing, as it does, the diagnostic value, pointed out by Duchenne, of determining the seat of the disease by the current, the indirect current or farad zation being powerless when the paralysis is of peripheral origin. As he improved the muscles gradually responded to the secondary current.

